# Phosphoinositide 3-Kinase-Dependent Signalling Pathways in Cutaneous Squamous Cell Carcinomas

**DOI:** 10.3390/cancers9070086

**Published:** 2017-07-11

**Authors:** Joanna M. Janus, Ryan F. L. O’Shaughnessy, Catherine A. Harwood, Tania Maffucci

**Affiliations:** Centre for Cell Biology and Cutaneous Research, Blizard Institute, Barts and The London School of Medicine and Dentistry, Queen Mary University of London, London E1 2AT, UK; j.m.janus@qmul.ac.uk (J.M.J.); r.f.l.oshaughnessy@qmul.ac.uk (R.F.L.O.); caharwood@doctors.org.uk (C.A.H.)

**Keywords:** Akt, cutaneous squamous cell carcinoma, mTOR, PI3K

## Abstract

Cutaneous squamous cell carcinoma (cSCC) derives from keratinocytes in the epidermis and accounts for 15–20% of all cutaneous malignancies. Although it is usually curable by surgery, 5% of these tumours metastasise leading to poor prognosis mostly because of a lack of therapies and validated biomarkers. As the incidence rate is rising worldwide it has become increasingly important to better understand the mechanisms involved in cSCC development and progression in order to develop therapeutic strategies. Here we discuss some of the evidence indicating that activation of phosphoinositide 3-kinases (PI3Ks)-dependent signalling pathways (in particular the PI3Ks targets Akt and mTOR) has a key role in cSCC. We further discuss available data suggesting that inhibition of these pathways can be beneficial to counteract the disease. With the growing number of different inhibitors currently available, it would be important to further investigate the specific contribution of distinct components of the PI3Ks/Akt/mTOR pathways in order to identify the most promising molecular targets and the best strategy to inhibit cSCC.

## 1. Introduction

Keratinocyte carcinomas (KC), comprising basal cell carcinoma (BCC) and cutaneous squamous cell carcinoma (cSCC), are the main forms of non-melanoma skin cancers (NMSC). They represent one third of all malignancies [1,2] and are the most common malignancy in the UK [3]. In 2014, there were 131,772 cases of NMSC registered in the UK, although this is a significant underestimation as there are acknowledged problems of under-recording [4]. The crude incidence rate indicates 233 new NMSC cases for every 100,000 males and 176 for every 100,000 females [5]. A recent study reported that approximately 3.3 million people were treated for NMSC in USA in 2012 [6]. More worryingly, the incidence of NMSC has risen over the years and it is still rising worldwide [7,8,9,10,11,12]. For instance one study estimated that on average the incidence of NMSC has increased by 3–8% yearly among white populations in Australia, Canada, Europe, and the USA in the last 30 years [12]. Morbidity associated with NMSC is high and available treatments can be disfiguring and expensive. One study estimated that in 2008 the cost due to skin cancer was in the range of £106–112 million in England, with expected cost per case estimated at £889–1226 for NMSC (bottom-up and top-down approaches) [13].

Approximately 75–80% of KC are BCC and 18–20% are cSCC [7,14]. While BCC is usually a localised cancer, approximately 5% of cSCC are able to metastasise, usually to lymph nodes [2,15]. As a consequence of this, although 95% of cSCC are curable with surgical resection, it has been estimated that 20% of skin cancer deaths are attributable to cSCC [16]. Indeed the ability of cSCC to metastasise leads to a 3-year disease-free survival rate of 56% [17] and a five-year survival rate of 25% to 35% [18,19,20,21]. Such a poor prognosis is due to a lack of therapies for this subset of patients as currently there is no FDA-approved therapy with a specific indication for metastatic cSCC [22]. The development of therapies is further complicated by the fact that no molecular biomarkers that can predict disease behaviour or treatment response have been validated [22]. With the rising incidence of this disease, a better understanding of the biochemical pathways involved in cSCC development and progression is urgently needed in order to identify molecular targets and design drugs that can be beneficial to patients. 

## 2. The Epidermis

**Figure 1:**
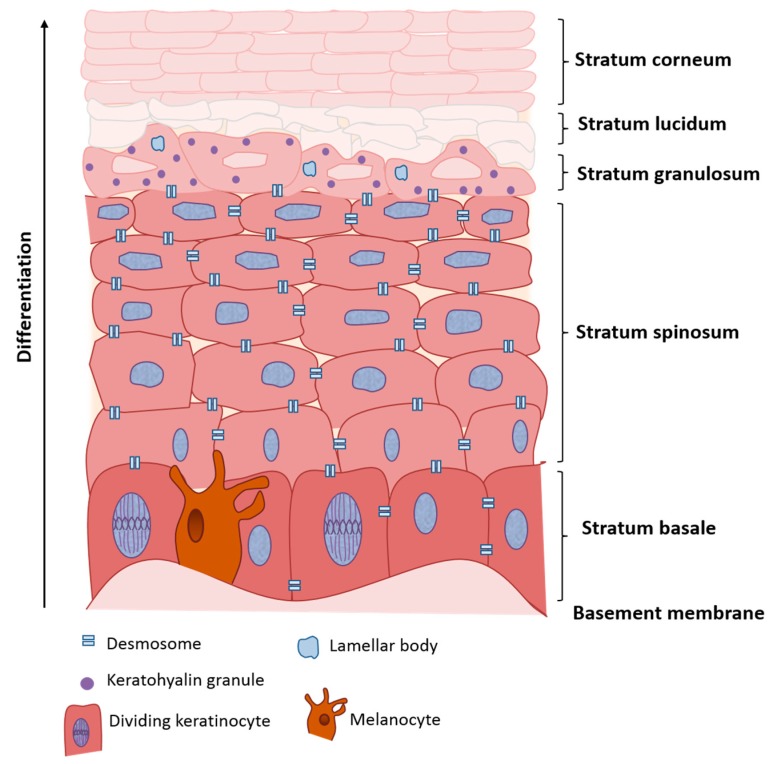
Representative diagram of the epidermis. The basement membrane, separating the dermis from the epidermis, and the distinct strata are indicated.

The epidermis of the skin contains stratified layers of squamous epithelium (Figure 1), mostly consisting of keratinocytes [23]. Keratinocytes are specialised cells named after their ability to produce keratin, a protein essential in the formation of intermediate filaments and in maintaining the barrier function of the skin. Keratinocytes continuously divide in the basal layer of the epidermis, and then differentiate as they migrate upwards through the spinous and granular layers towards the surface of the skin to ultimately form a layer of anucleate cornified cells called the stratum corneum [24,25,26]. As the cells migrate upwards they become more flattened and synthesise a number of different proteins (including different keratins) and lipids from specialised organelles, such as lamellar bodies and keratohyalin granules [27]. Intercellular junctions, such as desmosomes, are crucial to maintain the barrier function and modulate cell signalling [28]. The different desmosomal components have specific expression patterns within the epidermis and this is important to control not only the structure, but also the specific function of each stratum [29]. By the time they reach the surface the keratinocytes have become denucleated and form the tough keratinised layer of the stratum corneum, allowing the skin to remain waterproof and resistant to external stresses [23].

Keratinocytes that have left the basal layer of skin are squamous in morphology therefore they are generally referred to as squamous cells and are the most abundant cell type within the epidermis. KC are classified as BCC or cSCC depending on their histopathological characteristics; BCC cells tend to resemble those from the basal layer of the epidermis whilst cSCC tend to resemble the squamous cells [30]. 

## 3. Overview of cSCC Carcinogenesis

Chronic exposure to UV radiation has been described as the most important environmental risk factor for cSCC development, with other factors, including exposure to ionising agents and chemical carcinogens, also identified [31]. Indeed the majority of cSCC occurs on sun-exposed areas of the body and has been strongly associated with chronic sun exposure [32]. Approximately 65% of cSCC arise from dysplastic regions in the epidermis known as actinic keratoses (AK), which occur as a result of increased UV exposure [33]. The factors responsible for this progression are, however, still largely unknown and indeed not all AK progress to cSCC [34,35]. Nevertheless, AK are an important clinical risk factor for cSCC [36]. Genetically, cSCC is a very heterogeneous disease. Chromosomal changes have been identified by genome-wide studies, and mainly comprise loss of heterozygosity due to allelic loss and uniparental disomy at 3p, 9p, 2q, 8p and 13, and allelic gain on 3q and 8q [37,38]. Mutations in the *Notch* gene family and many other key genes, including *TP53*, have also been reported [39]. In fact, because of the complex mutational patterns, it is very difficult to identify driver genes in cSCC and this has strongly limited the translation from genomics to the clinic [39]. Indeed while identification of mutations in BRAF for advanced melanoma and Hedgehog signalling for BCC has paved the road to clinical use of BRAF and smoothened inhibitors respectively, a similar direct translation has not occurred in cSCC [39]. Nevertheless accumulating evidence from clinical use of epidermal growth factor receptor inhibitors or immune modulatory drugs suggests that targeted therapies may be beneficial [39,40]. There is, therefore, an urgent need to define the critical molecular mechanisms and key signalling pathways involved in cSCC carcinogenesis in order to identify new molecular targets.

It is now well documented that alteration of specific signalling pathways occurs during cSCC carcinogenesis. For instance reverse phase protein microarray analysis revealed specific activation of the mitogen-activated protein kinase (MAPK) pathway in cSCC compared to AK and normal skin [41]. Similarly, a core set of 196 genes was found to be differentially expressed between AK and cSCC and gene set enrichment analysis indicated a key role for MAPK pathway in cSCC compared to AK [42]. Consistent with this, more recently it has been shown that inhibition of MEK causes senescence, but not apoptosis, in cSCC cell lines and reduces tumour growth in vivo [43]. Several lines of evidence also indicate that activation of the enzymes belonging to the phosphoinositide 3-kinase (PI3K) family is involved in cSCC carcinogenesis (as discussed in more detail below).

## 4. The PI3K Pathway in Epidermal Homeostasis

PI3Ks catalyse the phosphorylation of position 3 within the inositol ring of specific phosphoinositides leading to the synthesis of lipid products that can then bind and mediate the activation of many signalling molecules [44,45,46,47]. Due to the ability of their products to activate many downstream effectors, PI3Ks have a well-established role in regulation of several cellular processes, including cell proliferation, growth, survival, migration, and metabolism [44,45,46,47]. Amongst the many enzymes that are regulated by PI3Ks, 3-phosphoinositide-dependent protein kinase 1 (PDK1) and protein kinase B/Akt are by far the most studied and well-characterised. Upon activation, binding of the PI3K product phosphatidylinositol 3,4,5-trisphosphate (PIP3) to Akt induces translocation of this enzyme to the plasma membrane where it can be activated through phosphorylation at its residue Thr308 by PDK1 and at residue Ser473 by additional kinases, including the complex 2 of mechanistic target of rapamycin (mTORC2) [48,49]. Activated Akt in turn regulates a plethora of signalling molecules, ultimately controlling cell proliferation, cell cycle, survival, and migration [50,51]. Three Akt isoforms exist, with data pointing to specific, non-redundant roles for each of them, in particular in cancer [52]. One of the key enzymes regulated by Akt is mTOR, a master kinase involved in protein synthesis, ribosome biogenesis, autophagy and several other cellular functions [53,54]. Activation of PI3K is normally tightly regulated and activation of PI3K-dependent pathways is also controlled by specific phosphatases, including the tumour suppressor phosphatase and tensin homolog (PTEN) which dephosphorylates PIP3 and switches off the signals [55].

PI3K-dependent pathways are crucial for regulation of epidermal homeostasis [56,57,58]. Data obtained through overexpression of constitutively active and dominant negative PI3K indicated a role of this pathway in the early phases of keratinocytes differentiation [56]. Consistent with this, it was reported that pan-PI3K inhibition induced premature differentiation of keratinocytes [57]. Activation of PI3K was indeed detected in mouse primary keratinocytes upon induction of differentiation and this was mirrored by activation of Akt [58], also confirmed by analysis of three-day old mouse skin that revealed increased active Akt in differentiating layers [58]. Activation of Akt has been associated with epidermal terminal differentiation with Akt1 in particular shown to be important for control of the barrier function of the cornified layer [59,60]. In this respect recent data have pointed to a role for Akt1 on nuclear degradation and differentiation through lamin A/C degradation [61]. Finally, data also indicate a role for PI3K in regulation of keratinocyte survival [62].

Transgenic mouse models have further supported a key role for PI3K-dependent pathways in epidermis. Mice bearing a keratinocyte-specific *PTEN* null mutation developed epidermal hyperplasia and hyperkeratosis [63]. A negative role for PTEN in regulation of skin growth was also confirmed in another study describing the phenotype of mice carrying a specific deletion of *PTEN* in the skin [64]. Additional evidence includes characterisation of a conditional PDK1 knockout model (with PDK1 ablated in activated CD4 T cells, regulatory T cells and mature keratinocytes) that revealed a central role for this enzyme in keratinocytes homeostasis [65]. Similarly, another study reported that epidermis-specific PDK1 knockout mice displayed a thin and shiny epidermis and impaired barrier function and pointed to a role for this enzyme in asymmetric cell division in the epithelium [66]. Finally, the Akt1/Akt2 null mouse lacks the stratum corneum and dies neonatally, possibly because of defects in the skin barrier [67].

Possibly the most compelling evidence of a key role for PI3K-dependent pathways in skin derives from the observation that germline mutations of *PTEN* lead to a number of severe disorders known as PTEN hamartoma tumour syndromes (PHTS) which are characterised by hyperplastic changes in the skin [68]. A typical example of PHTS is Cowden Syndrome, where most patients develop skin hamartomas and various skin lesions [68,69].

**Figure 2:**
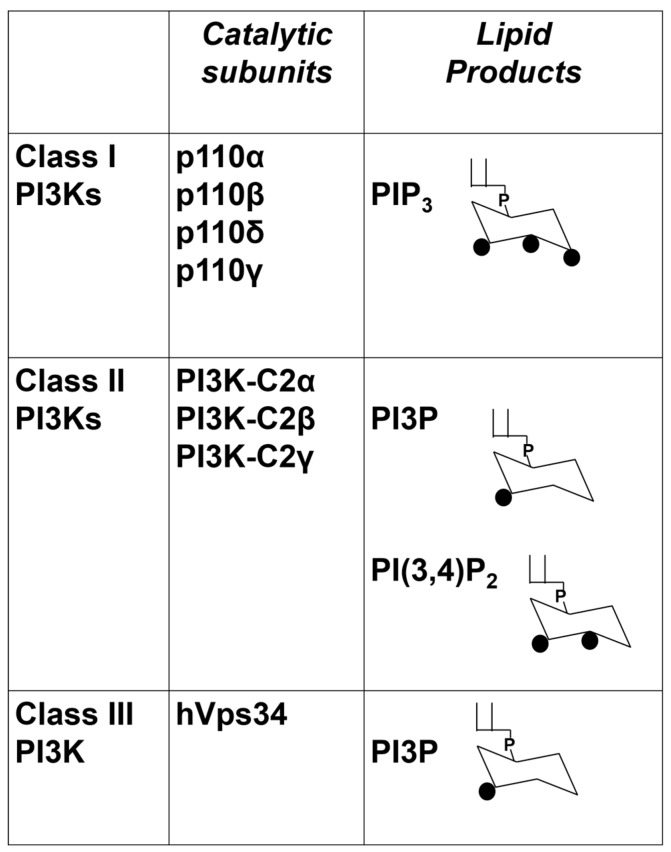
List of the eight mammalian PI3K isoforms and their classification into three distinct classes. For class I PI3Ks only the four catalytic subunits are shown. Their main lipid products are also indicated.

While evidence in literature has demonstrated the importance of the family of PI3Ks and corresponding PI3Ks-dependent pathways, less attention has been paid to the fact that eight distinct PI3K isoforms exist which are grouped into three classes according to their structures and substrate specificity [45,46,70,71], as depicted in Figure 2. Class I PI3Ks are dimers comprising a catalytic and a regulatory subunit and they catalyse the synthesis of PIP3 in vivo. Class II PI3Ks are monomers that mainly catalyse the synthesis of phosphatidylinositol 3-phosphate (PI3P) in vivo although evidence also indicates that they can catalyse the synthesis of phosphatidylinositol 3,4-bisphosphate (PI(3,4)P2). Class III PI3K only catalyses the synthesis of PI3P [46,71]. Isoform specific knock-out and knock-in mice and the investigation of the effects of isoform-specific inhibitors have shed much light on our knowledge of the physiological roles and the cellular functions that are regulated by each PI3K. It is now well established that these enzymes are not redundant and play distinct roles [72,73,74], but few studies have investigated the potential contribution of each of the eight PI3K isoforms to normal skin homeostasis.

Expression of the class I PI3K catalytic subunits p110α and p110β was detected in mouse epidermis and in cultured murine keratinocytes [57]. Ribonucleotide protection assays also revealed the presence of a transcript encoding the class I isoform p110γ in murine skin although the protein could not be detected [57]. Interestingly, upregulation of p110γ both at the mRNA and protein levels was observed during wound repair, in particular during the inflammatory phase [57]. Analysis of three-day old mouse skin revealed a specific localisation of the class I regulatory subunit p85α at cell-cell contacts of suprabasal differentiating keratinocytes [58]. Expression of two members of the class II subfamily of PI3Ks has also been reported in human epidermis, with PI3K-C2α found to be expressed throughout the epidermis and PI3K-C2β mainly restricted to suprabasal layers [75]. To the best of our knowledge no study so far has specifically investigated the expression levels and localisation of the class III PI3K hVps34 in the epidermis. In this respect it is worth mentioning that a recent study reported that autophagy is important during epidermal development and differentiation [76]. Due to the role of hVps34 in regulation of autophagy [77] it would be important to investigate the potential contribution of this PI3K isoform to skin homeostasis. 

A transient upregulation of p110α and p110β was detected in differentiating primary human keratinocytes in vitro [57]. Similarly, treatment of cultured human keratinocytes with calcium induced phosphorylation of p85α as well as activation of all class I PI3K isoforms, as assessed by in vitro assays [78]. Another study however showed that overexpression of either dominant negative p85 mutant (Δp85) or constitutively active p110α (p110α CAAX) did not induce differentiation of primary human keratinocytes, as assessed by Western blotting analysis of involucrin expression levels [75]. These authors further showed that overexpression of the class II PI3K-C2β, but not PI3K-C2α, was able to induce differentiation of primary human keratinocytes in vitro, although downregulation of these enzymes, either alone or in combination, did not appear to affect their calcium-induced differentiation [75]. Importantly, no difference in epidermal differentiation was detected in transgenic mice with either increased or absent PI3K-C2β expression, ruling out a major role for this enzyme in this process in vivo [75].

Evidence suggests that deregulation of PI3Ks-dependent pathways (possibly of specific PI3Ks-dependent pathways) can lead to alteration of the normal differentiation pattern and normal skin organisation. For instance, it was shown that stable overexpression of an inducible, constitutively-active mutant of p110α enhanced keratinocyte proliferation and migration, delayed differentiation in human keratinocytes and induced formation of disorganised, hyperplastic epithelium in organotypic skin cultures [57]. Selective roles for p110α or p110β were also reported in a transgenic mouse model which develops dermal lesions resembling PHTS [69]. By using mice lacking *PTEN* in epidermal keratinocytes (PTEN^Δ^) and mice with concurrent ablation of either p110α or p110β or both PI3K isoforms, the authors showed that p110α mainly regulated survival of suprabasal keratinocytes while p110β mainly regulated proliferation of basal keratinocytes in such a context of *PTEN* loss. A similar distinct regulation of Akt activation in the two layers was also observed in these transgenic mice [69]. Importantly, while PTEN^Δ^ mice developed multiple cutaneous hamartomas, concurrent ablation of either p110α or p110β significantly delayed both the development and severity of these skin lesions and simultaneous ablation of both PI3K isoforms completely prevented their development [69]. Relative mRNA levels of p110α and p110β were higher in cells from suprabasal and basal layers, respectively, and this was observed in cells from ear epidermis of both PTEN^Δ^ and wild-type mice, possibly suggesting a different role of the two isoforms also in normal skin epidermis.

Further studies are required to better define the contribution of each PI3K isoform in normal skin homeostasis and whether selective deregulation of some of them is associated with skin diseases. Improved understanding of the specific signalling pathways regulated by the distinct enzymes would also provide important information. For instance, although Akt undoubtedly plays a crucial role, it is very likely that PI3Ks mediate epidermal homeostasis via a number of different signalling pathways. Induction of PI3K signalling in the epidermis led to changes in expression of over 100 genes, with many associated with cell motility and adhesion as well as cell cycle control and DNA repair [57]. PI3K signalling has also been shown to inhibit the activity of the integrin-regulated YAP1 protein which is involved in epithelial cell proliferation [79]. Defining the contribution of the distinct isoforms could shed new light into the specific signalling pathways that these enzymes can control in epidermis.

## 5. PI3Ks-Dependent Pathways upon UV Irradiation

UV radiation causes DNA damage, for instance through generation of cyclobutane pyrimidine dimers (CPD) [80,81]. CPD have been associated with initiation of UVB-induced skin carcinogenesis [82] and repair or reduction of CPD in UVB-exposed murine skin reduces the risk of tumour development [83]. The nucleotide excision repair (NER) pathway is one of the mechanisms involved in the repair of UV-induced DNA damage [84]. It has been demonstrated that PTEN is necessary for efficient NER through regulation of the xeroderma pigmentosum proteins [85] and, therefore, alteration of its expression levels and/or function (and consequent deregulation of PI3Ks-dependent pathways) can lead to impaired DNA repair upon UV exposure. Indeed mice lacking PTEN in their epidermis are predisposed to skin tumourigenesis upon exposure to low sub-erythemal UV radiation [86]. UV radiation can induce alteration of PTEN levels/function through genetic alteration of the gene [87] or possibly through inactivation of the enzyme by UV-induced reactive oxygen species [88]. Indeed, reduced expression levels of PTEN were detected in transformed human keratinocytes upon chronic exposure to UVA radiation [89]. Similarly, it was shown that UVB radiation reduced PTEN levels in primary human keratinocytes, HaCaT keratinocytes and in mouse skin and this was associated with increased survival [90]. These authors further showed that downregulation of PTEN occurred at the transcriptional level and it was mediated by UVB-dependent activation of ERK and Akt [90]. Alteration of the PI3K pathway can also occur as consequence of alteration in the microRNA profile upon exposure to UV as observed in a study on SKH-1 hairless mice [91]. Consistent with the detected alteration of PTEN, several lines of evidence indicate that the PI3Ks/Akt/mTOR pathway is activated upon exposure to UV radiation. Phosphorylation of Akt [92,93] and mTOR [93] was reported in HaCaT cells treated with low doses of UVB as well as in SKH-1 mice treated with an acute dose of solar-simulated light (SSL) [94]. Moreover activation of Akt and mTOR was detected in sun-protected human skin after acute doses of physiologically-relevant SSL exposure [95]. Interestingly, one study reported differential regulation of Akt phosphorylation by UV, with phosphorylation of Ser473 mainly mediated by UVB and phosphorylation of Thr308 mediated by UVA in normal human epidermal keratinocytes [96]. On the other hand, both UV types were able to activate mTOR, as assessed by phosphorylation of S6K [96]. As UV represents the most important environmental risk factor for cSCC [39], it would be important to define the specific contribution of PI3Ks-dependent pathways, and in particular of the selective PI3K isoforms, on UV-driven cSCC carcinogenesis. 

## 6. PI3Ks-Dependent Pathways in cSCC

Deregulation of the PI3Ks/Akt/mTOR pathway is one of the most common mechanisms responsible for development and progression of many cancer types [97,98,99,100]. Reverse phase protein microarray analysis revealed activation of a number of key proteins involved in this pathway in advanced and non-advanced human cSCC compared to AK [41]. Constitutive activation of the Akt/mTOR pathway in epidermal tumours was also reported in another study, with levels of phosphorylated Akt and mTOR shown to be much higher in 15 samples of SCC than in the same number of normal or AK skin samples [101]. Moderate/strong phosphorylation of Akt at Ser473 was also detected in 10 out of 15 cSCC and in eight out of 10 metastatic cSCC [101]. A specific role for distinct Akt isoforms has also been suggested by the observation that down-regulation of Akt1 and upregulation of Akt2 occur commonly in cSCC [102]. In addition, activation of upregulated Akt2 is associated with high-grade tumours [102].

Some studies have investigated the mechanisms responsible for activation of PI3Ks-dependent pathways in cSCC. Activating mutations of *PIK3CA*, a common characteristic of many cancer types, including lung SCC and head and neck SCC (HNSCC), have been reported but do not appear to occur at high frequency in cSCC [103]. For instance whole exome sequencing on DNA from 39 patients reported that *PIK3CA* was mutated only five times in four patients and, importantly, none of these mutations were the “classical” hotspot mutations observed in other tumour types [104]. On the other hand, a more recent study of 122 recurrent, metastatic cSCC identified clinically-relevant genomic alterations of *PIK3CA* in 6% of the cases [105]. This was consistent with data from a cohort of metastatic cSCC (29 cSCC lymph node metastases) that identified a *PIK3CA* P471L mutation in some of these tumours [22]. Importantly, a sustained clinical response was observed in one patient with metastatic cSCC harbouring mutations (including the *PIK3CA* P471L mutation) upon treatment with the mTOR inhibitor temsirolimus [105]. It remains to be established whether this mutation is indeed associated with hyperactivation of PI3K-dependent pathways. It is worth mentioning that this specific mutation was also detected in one primary cSCC sample [106], possibly suggesting that this event might not be specifically associated with metastatic cSCC although additional studies would be required to confirm this observation. A few additional mutations in other PI3K isoforms were observed in this same study [106], although the limited number of specimens does not allow the drawing of any conclusions about their importance and relevance. 

Loss of PTEN function is a common mechanism responsible for hyperactivation of PI3Ks-dependent pathways in many cancer types. Although somatic mutations of *PTEN* are rare in skin lesions, reduced levels of PTEN have been detected in human AK and cSCC, indicating that either epigenetic modifications or post-transcriptional downregulation of PTEN might be involved in the progression of the disease. Indeed, while initial studies did not detect any deletion (47 cSCC) [107] or somatic mutations (21 cSCC) [108] or hypermethylation of the promoter (20 cSCC) of *PTEN* [109], a more recent study showed that loss of protein expression of PTEN was observed in 15 out of 16 cSCC and this was associated with an increase in fibroblast growth factor 10, which in turn plays a central role in cSCC promotion [110]. Some mechanisms that can lead to inactivation/loss of PTEN have been observed in animal models. For instance it has been shown that loss of protein expression of PTEN can occur upon genetic ablation of the developmental transcription factor *grainy head-like 3*. This is associated with activation of the PI3K pathway and formation of aggressive cSCC which are completely inhibited by restoration of PTEN [111]. Finally, PTEN alteration can occur as a result of UV exposure, as discussed above. Alternative mechanisms to PTEN alteration, ultimately leading to hyperactivation of PI3Ks-dependent pathways, might also exist in the context of cSCC. For instance increased formation of spontaneous precancerous lesions and cSCC was reported in transgenic mice expressing the tyrosine kinase Fyn (K14-Fyn Y528F mice) together with increased activation of several signalling pathways, including increased phosphorylation of PDK1 [112]. PI3K/Akt activation has also been detected downstream of the basement membrane proteins laminin-332/collagen VII and proved to be crucial in mediating their contribution to cSCC tumourigenesis and invasion [113].

The impact of activation of PI3Ks-dependent pathways on cSCC development and progression has been demonstrated in many studies using transgenic animal models. Conditional knockout of PTEN in skin induces neoplasia and is critical for skin cancer development [64,88]. Analysis of transgenic mice bearing a *PTEN* null mutation specifically in the keratinocytes revealed that 100% of these mice developed spontaneous tumours within 8.5 months of birth, mostly squamous papillomas [63]. Importantly, many of these papillomas further developed into SCCs which were able to invade the dermis. In addition, the keratinocyte-specific *PTEN* ablation resulted in accelerated tumourigenesis upon chemical treatment [63]. Analysis of mouse skin tumours showed that PTEN was detectable in differentiating areas of the papilloma and in the most differentiating areas of cSCC whereas it was undetectable in non-differentiating infiltrative areas of cSCC [114]. Models of mouse skin tumourigenesis further demonstrated the central role for PI3K/Akt during both tumour formation and progression stages. Evidence includes demonstration of the critical role for Akt in insulin like growth factor-1 (IGF-1)-mediated mouse skin tumour promotion [115,116]. An increase in Akt activity was also detected throughout the entire process in the two-stage model of mouse skin carcinogenesis [114] and overexpression of Akt in mouse primary basal keratinocytes accelerated tumourigenesis upon injection into mice [114]. Furthermore transgenic mice expressing increased levels of Akt or constitutively-active Akt in the basal layer of stratified epithelia displayed higher sensitivity to the tumour promoter 12-*O*-tetradecanoylphorbol-13-acetate and increased sensitivity to two-stage skin carcinogenesis [117]. 

The specific mechanisms by which PI3Ks/Akt regulates cSCC promotion involve both increased cell proliferation and resistance to apoptosis, as detected in PTEN-deficient keratinocytes [63]. Similarly, the pathway has been implicated in resistance to apoptosis mediated by the receptor tyrosine kinase Axl in cSCC [118]. Interestingly, it has been recently demonstrated that Axl is involved in development of resistance to a class I PI3K p110α inhibitor in HNSCC and in oesophageal SCC (OSCC) [119], suggesting a complex interplay between the Axl-dependent and PI3Ks-dependent signalling pathways in SCC.

## 7. Targeting PI3Ks-Dependent Pathways in cSCC

**Figure 3:**
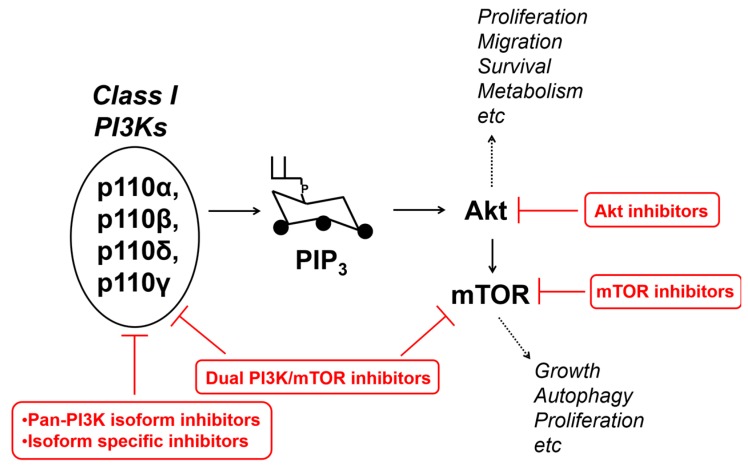
Schematic and simplified representation of the class I PI3Ks/Akt/mTOR pathway, some of the main cellular functions regulated by it, and the main family of inhibitors targeting it.

The PI3Ks/Akt/mTOR pathway is a well-established target for anti-cancer drugs development [97,98,120,121,122,123,124,125] and several inhibitors have been developed, targeting PI3K, Akt, mTOR, as represented very schematically in Figure 3. As for the class I subfamily, several inhibitors are currently available, including inhibitors that target all isoforms with similar IC_50_ (pan-PI3K) or mainly one/more-than-one selective isoforms (isoform-specific, i.e., with a much lower IC_50_ towards one/more-than-one isoforms compared to the others) [125]. Isoform-sparing PI3K inhibitors have also been developed, as is the case of GDC-0032, an inhibitor showing much less potency towards p110β (β-sparing) [126]. Finally, dual PI3K/mTOR inhibitors have also been developed [125].

Isoform-specific PI3K inhibitors were developed with the aim of reducing side-effects and increasing potency, by specifically targeting the main isoform(s) involved in the development/progression of each specific cancer type [124,125]. For instance this led to trials of p110α inhibitors in cancers harbouring activating *PIK3CA* mutations or p110β inhibitors in tumours driven by PTEN loss, as this specific isoform was reported to be critical in this context [127,128,129,130,131]. Similarly, due to their high expression in immune cells, inhibitors of p110δ and p110γ (or targeting both isoforms) have been tested in many haematological malignancies, with a selective p110δ inhibitor (Idelalisib) approved for use in chronic lymphocytic leukemia and follicular B-cell non-Hodgkin lymphoma [125]. With the increasing evidence suggesting the importance of the microenvironment for tumour development/progression, the potential beneficial effects of p110δ and p110γ inhibitors in other cancer settings are also being tested.

To the best of our knowledge no studies so far have reported results from clinical trials aimed to assess the effect of pan-PI3Ks or isoform-specific inhibitors in cSCC. On the other hand, these inhibitors have been tested or are being tested in other SCC [125]. For instance, as the PI3K pathway is the most frequently mutated pathway in HNSCC, several inhibitors have been or are being tested in this context, either alone or in combination with other interventions [132]. These include pan-PI3K inhibitors (Buparlisib (BMK120), PX-866, Copanlisib (BAY 80-6946), SF1126) and isoform-specific inhibitors (Alpelisib (BYL-719, NVP-BYL719) or the p110δ inhibitor AMG319), as well as Akt and mTOR inhibitors [132]. According to the clinicaltrials.gov website, at the time of writing this review, other trials are ongoing or are recruiting participants to test PI3K inhibitors in different SCC, including OSCC and squamous non-small cell lung cancer either alone or in combination with other drugs. 

Overall data in literature indicate that targeting the PI3Ks/Akt/mTOR pathway could be beneficial in cSCC [133]. For instance studies have demonstrated the beneficial effects of the mTOR inhibitor rapamycin in animal models, such as in mice receiving chronic sub-erythrogenic doses of UVB and UVA, where rapamycin increased latency of large tumours and reduced their multiplicity [134]. Decreased tumour multiplicity, size, and progression were also detected in hairless mice exposed to UVB upon treatment with rapamycin alone or in combination with cyclosporine [135]. Rapamycin also reduced tumour incidence and multiplicity in a chemically-induced mouse model [136]. Another study further reported that rapamycin reduced not only the tumour burden of mice harbouring early and advanced tumour lesions but also recurrent skin SCCs in a chemically-induced cancer model, basically resulting in regression of carcinogen-induced skin SCC [137]. More importantly, the beneficial effects of mTOR inhibitors towards cutaneous carcinogenesis have been observed in specific subsets of patients. Prolonged immunosuppression strongly increases the risk of cSCC in organ transplant recipients, with a 65–100 fold increased incidence observed in transplant recipients compared to the general population [138,139,140]. These cutaneous malignancies are also generally more aggressive and numerous than those seen in the general population [138,139]. A significantly reduced risk of developing post-transplant de novo malignancies and non-skin solid malignancy was observed in patients receiving mTOR inhibitors (sirolimus/everolimus) as immunosuppressants compared to patients receiving calcineurin inhibitors (CNI) [141]. Switching renal transplant recipients receiving CNI-based therapies to sirolimus resulted in reduced incidence of de novo KC formation [142,143,144,145] and even regression of pre-existing premalignant lesions [144]. While these data suggest a potential beneficial role for mTOR inhibitors, it is important to mention that in many cancer settings the use of some inhibitors of the PI3Ks-dependent pathways has unfortunately led to the discovery of compensatory mechanisms that reduce their therapeutic efficiency [146,147]. One of the most characterised mechanisms of resistance was identified through the use of mTOR inhibitors that were reported to induce hyperactivation of Akt through removal of a negative feedback loop [148,149,150]. Increased Akt phosphorylation upon treatment with rapamycin has also been observed in keratinocytes, confirming the existence of such a feedback loop in these cells [60]. Possibly consistent with this, a study in SKH-1 mice reported that while rapamycin indeed reduced tumourigenesis when it was applied topically after mice were exposed for 15 weeks to SSL, tumourigenesis was actually increased if rapamycin was applied during SSL exposure and for an additional 10 weeks [94]. Importantly this study further showed that the selective PDK1/Akt inhibitor PHT-427 was able to prevent this latter effect, indicating that combination of drugs targeting distinct components of the PI3Ks-dependent pathways could prevent or oppose potential compensatory mechanisms [94].

The question remains as to whether targeting PI3Ks directly using either pan-PI3Ks or isoform-specific inhibitors would represent a valid therapeutic option in cSCC. It was previously shown that inhibition of PI3Ks with the pan inhibitor LY294002 reduced chemically-induced skin tumour promotion in a mouse model overexpressing IGF1 [116]. Additionally, selective simultaneous inhibition of p110α and p110β not only prevented the development of PHTS in mice lacking *PTEN* in epidermal keratinocytes (PTEN^Δ^) but it was also able to reverse advanced skin hamartomas [69]. With the increasing number of PI3Ks inhibitors currently available, an improved understanding of the relative contribution of each isoform in cSCC carcinogenesis, in particular in the context of metastatic cSCC, would be useful to ascertain the potential impact of these drugs.

## 8. Conclusions

Despite several data indicating that PI3Ks-dependent signalling pathways are important in cSCC much still needs to be understood about the contribution of these enzymes and, in particular, the selective contribution of each of the distinct PI3K isoforms to the disease. Currently, the lack of strong evidence indicating either specific mutations or selective activation of specific PI3K isoform(s) during cSCC carcinogenesis, in particular during progression to metastatic cSCC, makes it difficult to envisage which selective PI3K inhibitor(s) or which specific drugs combination(s) could be beneficial in this context. Additional investigations, including a better characterisation of the role of distinct PI3Ks, are needed to determine whether targeting selective PI3Ks could represent a useful strategy to counteract this disease, in particular for metastatic cSCC.
